# The treatment of cervical intraepithelial neoplasia grade 2 (HSIL): between active surveillance and surgery—a 10-year monocentric data analysis

**DOI:** 10.1007/s00404-025-08097-1

**Published:** 2025-07-08

**Authors:** Ulrike Ehlers, Lars Hoischen, Jan Lennart Stalp, Jens Hachenberg, Dhanya Ramachandran, Bianca Brüning, Matthias Jentschke, Peter Hillemanns, Agnieszka Denecke

**Affiliations:** 1 Department of Obstetrics and Gynecology, Hannover Medical School, Hanover, Germany; 2 Department of Sociology, Leibniz University, Hannover, Germany

**Keywords:** CIN 2, HPV Infection, Treatment

## Abstract

**Purpose:**

In recent years, active surveillance has been introduced as an alternative to excisional treatment in younger women with cervical intraepithelial neoplasia grade 2 (CIN 2) because spontaneous regression rate is high and excisional treatment is associated with an increased risk of preterm birth. However, the potential effect of this conservative approach on the risk of developing cervical cancer has not been evaluated very well.

**Methods:**

The present study offers a real-life analysis of treatment pathways for patients diagnosed with CIN 2.

**Results:**

Following CIN 2 diagnosis, 84 cases out of 187 (44.9%) were managed conservatively for at least 7 months and 103 cases (55.1%) were admitted for an excisional procedure LEEP (loop electrosurgical excision procedure). Out of 84 patients observed with a CIN 2 diagnosis, 64 showed persistence of CIN 2 lesion (76.2%), 14 showed spontaneous remission under active surveillance (16.7%), 4 progressed to CIN 3 (4.8%) and 2 to carcinoma (one case of vaginal carcinoma and one case of cervical adenocarcinoma (Supplementary Fig. 1) (2.4%). We observed the regression of CIN 2 in 16.7% of patients on active surveillance and this was statistically significant during the observation period (95% CI 5.72–10.85, *p* < 0.001) (Supplementary Fig. 3).

**Conclusion:**

The choice of treatment was strongly associated with HPV typing in our study. Patients with HPV 16 infection underwent surgery more often than patients without HPV 16 infection. The difference in our study was statistically significant (*p* < 0.001). We observed regression of CIN 2 in 16.7% of patients on active surveillance and this was statistically significant to the observation period (95% CI 5.72–10.85, *p* < 0.001).

**Supplementary Information:**

The online version contains supplementary material available at 10.1007/s00404-025-08097-1.

## What's new

**Table:** 

A statistically significant correlation between HPV 16 positivity and the choice of surgical therapy was observed. HPV-subtyping and p 16 positivity can simplify the selection of treatment.	

## Introduction

One of the primary objectives of cervical cancer screening is to prevent the development of cervical cancer by facilitating the early detection and treatment of precancerous lesions [1]. Persistent infection by high-risk human papillomavirus (hr-HPV) infection is known to exacerbate cancer development with known environmental and genomic risk factors [2].

Cervical intraepithelial neoplasia (CIN) lesions are traditionally classified according to their histological characteristics and the associated prognosis. The low-grade lesions (LSIL and CIN 1) represent an active and transient HPV infection, with a relatively low rate of progression to cervical cancer. In contrast, high-grade lesions (HSIL, CIN 2, and CIN 3) are associated with a higher risk of progression to cervical cancer [3].

Cervical intraepithelial neoplasia grade 2 (CIN2) is a precursor of cervical cancer and may progress if left untreated [4]. The management of CIN 2 with immediate Loop electrosurgical excision procedure (LEEP) has been debated within the past years as studies have shown high spontaneous regression rates (50–60% within 2 years) of CIN 2 indicating overtreatment. There is clear evidence that patients with conservative observation and follow-up examinations can achieve a good spontaneous regression rate. Tainio et al. described spontaneous regression rates of 50–60% during the following two years. A CIN1 lesion has a 60% chance of regressing, while a CIN2 lesion has a 40% chance and a CIN3 lesion has a 33% chance [5, 6]. Although WHO guidelines recommend treating high-grade lesions (CIN 2/3), it is also acceptable to observe patients with CIN 2 to avoid the morbidity linked to operative treatment. In recent years, active surveillance has been introduced in many countries as an alternative to excisional treatment [7, 8].

Depending on the transformation zone, the current German S3 guideline recommends re-examination in 6 months for transformation zone type I. For transformation zone type II and III, further histopathological clarification with endocervical curettage (eC) should be performed if CIN 2 is confirmed. In patients under 51 years of age with transformation zone types I and II and no discrepancy between colposcopy, histology, and cytology, surgical treatment in the form of laser vaporization could also be performed. The only approved treatment for CIN 2 in transformation zone types II and III is an excision of the cervical transformation zone. This treatment carries some complications, including intraoperative bleeding and an increased risk of preterm delivery in future pregnancies. Long-term active surveillance of CIN 2 has only been recommended for younger women (under 25 years) and pregnant women. As active surveillance is based on follow–up with colposcopy and collection of colposcopies directed cervical biopsies, women undergoing active surveillance may be at a higher risk of prevalent disease, including cervical cancer [9].

In contrast to the German guideline, the American recommendation of the ASCP favors the excisional approach. For nonpregnant patients 25 years or older, expedited treatment, defined as treatment without preceding colposcopic biopsy demonstrating CIN 2 + , is preferred when the immediate risk of CIN 3 + is ≥ 60%, and is acceptable for those with risks between 25 and 60%. The expedited treatment is preferred for nonpregnant patients 25 years or older with HSIL cytology and concurrent positive testing for HPV genotype 16 (HPV 16) (HPV 16-positive HSIL cytology) and never or rarely screened patients with HPV-positive HSIL cytology regardless of HPV genotype. Excisional treatment is preferred to ablative treatment or active surveillance for histologic HSIL (CIN 2 or CIN 3). Recommendations of colposcopy, treatment, or surveillance of CIN 2 based on a patient’s risk of CIN 3 + determined by a combination of current results and past history [10].

Conservative therapy is not evidence-based and requires greater experience on the part of the treating clinician. It is known that women who have had CIN 3 removed have an increased risk of developing cervical cancer [11, 12]. The risk of progression of CIN 2 lesions into CIN 3 is 20%, and further progression into cervical cancer is 5%. CIN 3 has a 12% rate of progression into invasive disease, making treatment necessary in most cases. [13, 14].

In a large Danish registry study with over 27,000 women between 18 and 40 years of age in 2023, Lycke et al. demonstrated that the cumulative risk of developing cervical carcinoma was significantly higher in the observation group compared to women who received surgical treatment in the observation group [15]. This study shows that the cumulative risk of cervical carcinoma in persons with conservative therapy/observation is 0.56%. This is significantly higher than the figures reported in other studies and systematic meta-analyses, which found a risk of between 0.33 and 0.47% [16]. Overall, the absolute risk of cervical cancer in patients with a history of CIN 2 is low at 0.8–7% [17]. Thereafter, active surveillance was associated with nearly four-fold higher risk of cervical cancer compared with immediate LEEP [18].

An interesting aspect is disease progression and the need for surgical therapy during the follow up. It has been reported that 40–50% of women will experience progression or persistent disease requiring excisional therapy over time [19–21]. There is a clear need to gather evidence on this important topic.

The present study offers an analysis of the treatment pathways for patients diagnosed with CIN 2 at a university hospital with a dedicated dysplasia unit. Our real-world data compare the outcomes of active surveillance versus excisional treatment for CIN 2 lesions in clinical practice. We further identify the risk factors for possible persistence and progression to CIN 3 + lesions in the group of patients under observation.

## Methods

### Ethics approval

This study was approved by the ethics committee of Lower Saxony in Hannover (11583-BO-K-2024). Written consent was obtained from all the participants including an agreement that pseudonymized data may be used for research. The handling and publication of patient data in this study were performed strictly in accordance with the Declaration of Helsinki DoH/Oct 2008 and included confidentiality and anonymity.

### Patient cohort and inclusion criteria

We retrospectively analyzed data from 187 patients diagnosed with cervical intraepithelial neoplasia grade 2 (CIN 2, HSIL) referred to the dysplasia unit at Hannover Medical School between 2013 and 2024. The inclusion criteria were histologically confirmed CIN 2 (HSIL) regardless of the patient’s age, cytology, HPV status—or type of transformation zone (TZ) (Supplementary Fig. 1). The duration of observation and the resulting outcome were contingent upon the temporal parameters of observation. The patients in the observation arm were examined every 6 months in the dysplasia unit. The examination included anamnesis, colposcopic examination, HPV testing and cytological smears. In the case of major changes, a colposcopy-guided biopsy was taken. In addition, we tested whether the age of the patients at the time of diagnosis was associated with progression to carcinoma in this period. All conizations were performed by surgeons with colposcopy experience. Conizations were performed under colposcopic control and exclusively as LEEP procedure. Endocervical biopsy was part of the operative procedure and was performed in all patients to exclude the endocervical lesions.

### Histological analyses

Conventional Pap smear was done routinely before colposcopy and reported according to the Munich nomenclature 3. For study purposes we transferred the Munich nomenclature to the 2015 Bethesda System for Reporting Cervical cytology including terminologies as—negative for intraepithelial lesion of malignancy (NILM), atypical squamous cells which is further subdivided into atypical squamous cells of undetermined significance (ASC-US) and atypical squamous cells, cannot exclude high-grade squamous intraepithelial lesion (ASC-H), low-grade squamous intraepithelial lesion (LSIL), high-grade squamous intraepithelial lesion (HSIL) and /or squamous cervical carcinoma (SCC). We used the Rio 2011 nomenclature for colposcopy description.

### Colposcopy analyses

All patients were examined in the clinic by colposcopists trained according to the German Society for Colposcopy and Cervical Pathology (AG-CPC) before operative treatment was indicated a colposcopy-directed forceps biopsy was taken from the patients based on colposcopically confirmed major changes. Only data from patients with histologically confirmed CIN 2 (HSIL) were included in the final analysis.

### HPV status determination

HPV tests were carried out using either the Aptima HPV assay (Roche Molecular Diagnostics, Laval, Quebec, Canada) or the COBAS method (Roche Diagnostics, Mannheim, Germany) [22].

### Statistical analysis

The data were analyzed using SPSS version 28.0 (SPSS Inc., Chicago, IL, USA). Descriptive statistical analysis, Spearman and Pearson correlation analyses and chi-square tests were employed. Continuous data were shown as mean ± standard deviation. The categorical data were presented as counts and proportions. A *p* value below 0.05 was considered to indicate statistical significance.

## Results

### Study population and management of CIN 2

We analyzed data from 187 patients diagnosed with cervical intraepithelial neoplasia grade 2 (CIN 2, HSIL). The patients were referred to the dysplasia consultation between 2013 and 2024. The median age of our patient collective was 40.9 years. 103 (55.1%) patients with CIN 2 underwent operative treatment (LEEP). A sub-group of 84 patients underwent active surveillance (44.9%) (Supplementary Fig. 2, Table 1). The primary reason for undergoing observation in lieu of operation was reported to be unfinished family planning and the patient’s desire to avoid surgery. The mean duration of observation amounted to 15 months. 7 patients were observed over a period of 5 years (60 months). In the entire patient collective, conservative management was carried out in eight patients with more than 60 months after the medical diagnosis of CIN 2. (Supplementary Fig. 3).

**Table 1:** Duration of observation in the active surveillance group

Duration of observation (months	Prevalence (number of patients)	Percentage
6	24	16.9%
12	26	13.4%
18	18	9.1%
24	7	3.5%
≥ 60	15	7.5%

Patients with HPV 16 infection underwent surgery more often than patients without HPV 16 infection. The difference was statistically significant (*p* < 0.001) (Table 2).

**Table 2:** HPV-genotyping distribution

Genotyping of HPV	Prevalence (number of patients)	Progression to CIN 3 (number of patients)	Regression (number of patients)
Single infection			
HPV 16	55	2	3
HPV 18	4	0	1
HPV OT	51	4	12

17.4% of patients in our cohort were smokers. The smoker sub-group was younger than the non-smoking sub-group in our study. However, smoking was not a significant risk factor for progression (*p* = 0.324) (Supplementary Fig. 1).

### HPV-infection

Persistent HPV-infection was confirmed in 80.5% of cases. The overall prevalence for high-risk HPV 16 was 55 and 4% for HPV 18 in our cohort (Supplementary Tables 1 and 2).

Our findings revealed that 30.3% of patients in our patient group had single infection. 28.2% of patients were infected with HPV 16 and 2.1% with HPV 18. Multiple infections occurred in 8.7% of cases, with HPV 16 and HPV 18 double positive accounting for a majority of this group (3 patients, 1.5%). We observed the occurrence of HPV 16 and HPV other types (OT) in 6.2% of cases (in 12 patients). In two patients (1.0%), we noted the presence of HPV 18 and OT.

### Cytology results in CIN 2 patients

Cytological results were at hand for 79.7% of the patients (*n* = 149). The most common results were LSIL cytology with Pap III D1 (24.6%) and HSIL cytology Pap III D2 (25.1%), which was consistent with the histological diagnosis. 15.0% of patients had Pap IV a-p (HSIL cytology) findings in our group (Table 3).

**Table 3:** Cytology results

Cytology	Bethesda system	Prevalence (number of patients)	Percentage (%)
Pap I	NILM	24	12.8
Pap II	ASC-US	4	2.1
Pap III D1	LSIL	46	24.6
Pap III D2	HSIL	47	25.1
Pap IV a-p	HSIL	28	15.0
cytology not available		38	20.3

### Progression and regression of CIN 2

We observed regression of CIN 2 in 14 cases (16.7%) in the patient collective under active surveillance and it was found to be statistically significant in comparison to operative treatment (*p* < 0.001, CI 5.72–10.85) (Supplementary Fig. 5). Within our two main groups (active surveillance vs primarily surgery), we saw a progression of 3.6% in the group under observation. Histological upgrading was noted after surgery in 20.4% cases suggesting that colposcopy frequently underestimates HSIL diagnoses, and the histopathological diagnosis of CIN 2 and differentiation from CIN 3 is complex (Supplementary Table 2).

An interesting aspect was the correlation of age with regressive changes. We find that with higher age, fewer regressions were observed in the active surveillance group (Supplementary Fig. 5).

Additionally, two patients with initial CIN 2 diagnosis were diagnosed with progression to cancer:

- 37-year-old nulligravida with diagnosis of CIN 2 and cervical polyp.

After 6 months of observation, conization was performed. The histological work-up revealed adenocarcinoma of the cervix in stage FIGO I (pT1a2). The patient underwent trachelectomy, laparoscopic pelvine sentinel lymphonodectomy and cerclage.

#### Picture 1

Pre-operative colposcopy
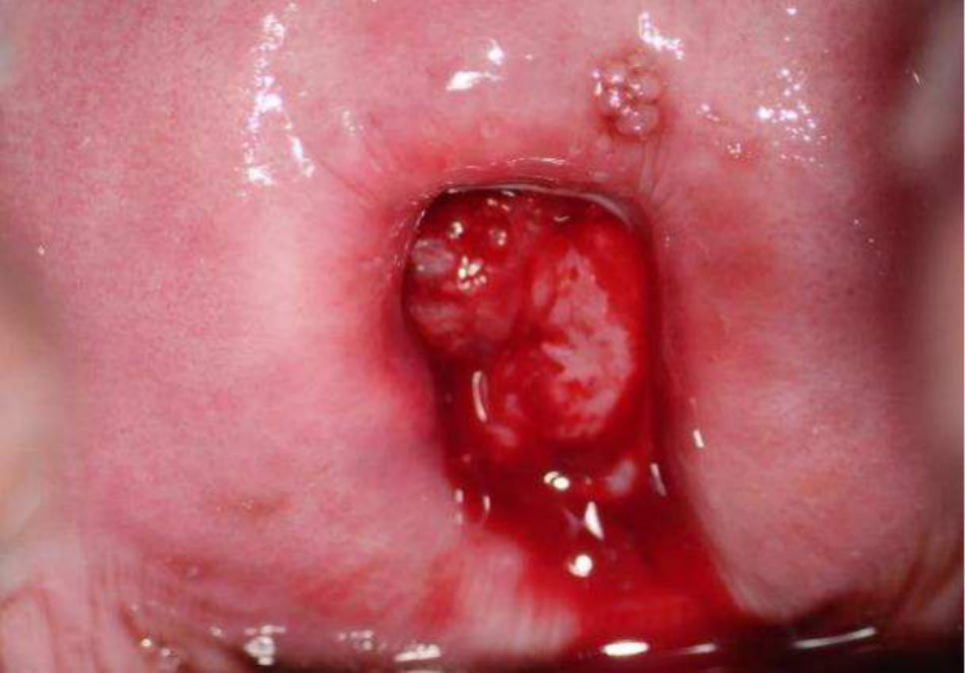

(b) The second patient, a 66-year-old woman with a carcinoma of the vagina was diagnosed in 2023. The patient underwent conization in 2017 due to CIN 2 and secondary diagnosis of HSIL (VaIN 2) of the vagina. The patient was followed for 60 months due to cytological abnormalities and renewed CIN 2 formation. In 2023, the patient was diagnosed with vaginal carcinoma. The subsequent colpectomy and conization revealed no abnormalities in the portio.

#### Picture 2

Cervical polyp (Type 3 transformation zone after conization and atrophy of vagina, 2022)
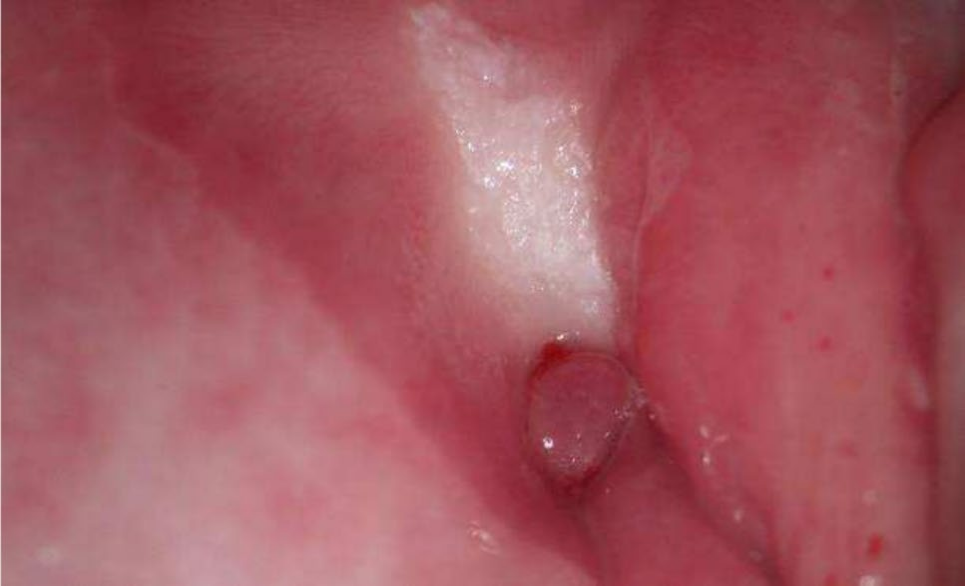


#### Picture 3

HSIL in the vaginal left wall (2022)
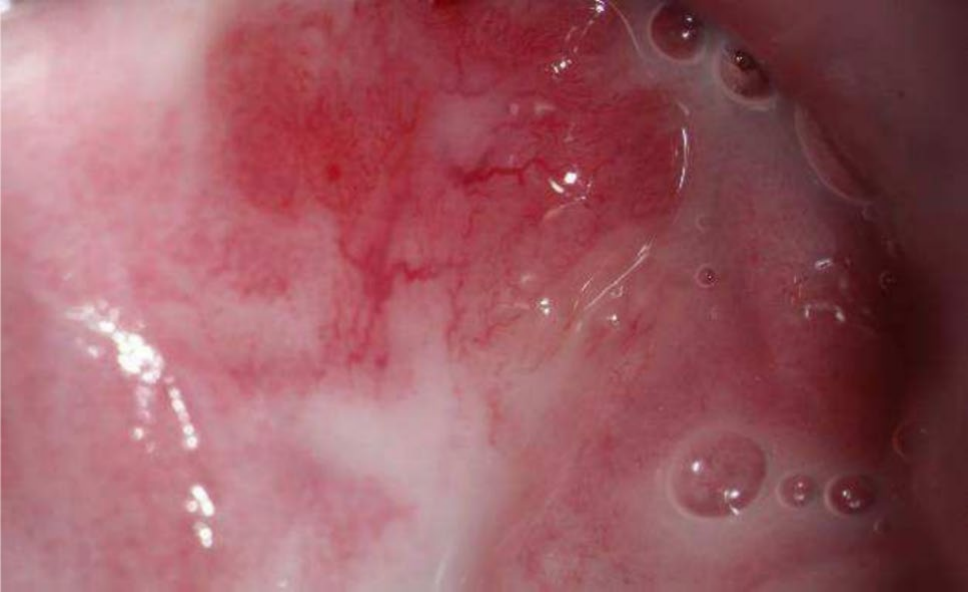


#### Picture 4

Vaginal carcinoma (2023)
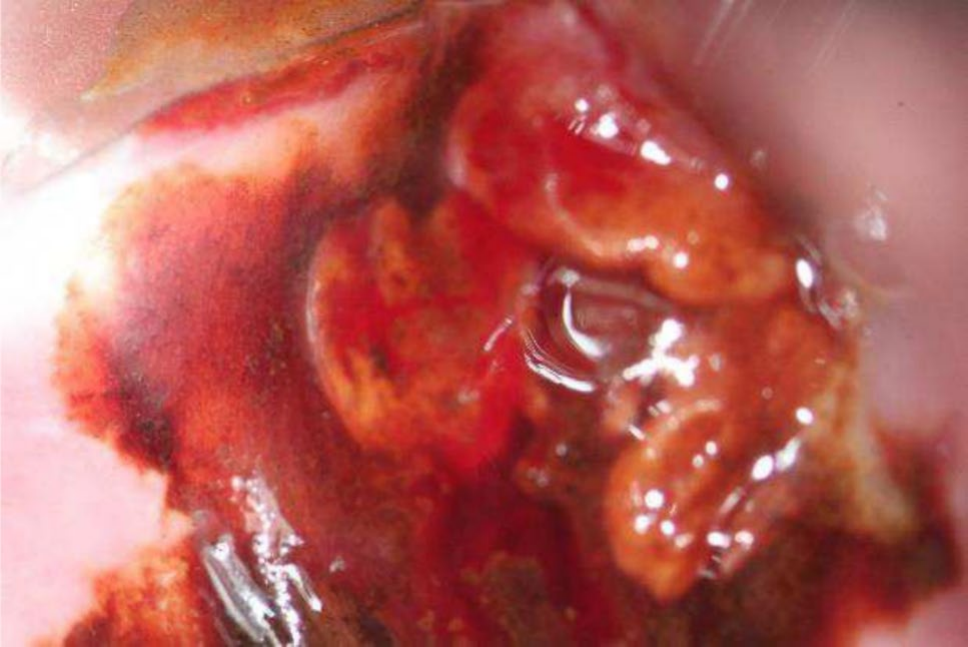


### Risk factors

Smoking was tested for association with progression or regression in our two groups. While it showed an influence on progression, the correlation was not statistically significant (*p* = 0.12, CI 8.35–14.36).

We also analyzed immunosuppressed patients. Immunosuppressed patients accounted for approximately 5.9% of our patient cohort. We found that HPV 16 positivity and immunosuppression correlated with progression in our study. However, this correlation was not statistically significant, likely due to the small group of immunosuppressed patients in our cohort (5.9%, *n* = 11, *p* = 0.53).

## Discussion

There is currently insufficient data-based evidence for the treatment of cervical lesions with CIN 2. The question that concerns us clinically is how high the chance of spontaneous regression is under active surveillance and which patients benefit from immediate surgical treatment. We were able to confirm that in 16.7% of cases, we saw spontaneous regression of the lesions during follow-up. Especially in younger patients, the spontaneous regression rate was up to 28.6%. This shows that treatment of CIN 2 lesions can lead to overtreatment and guideline-compliant monitoring is the best option for patients under the age of 35.

While observing the patients with moderate lesions, the progression to cancer was noteworthy. In general, there is a lack of data on the progression of CIN 2 to carcinoma in this patient group [23].The current German S3 guideline recommends re-examination in 6 months for transformation zone type I. For transformation zone type II and III, further histopathological clarification with endocervical curettage (eC) should be performed if CIN 2 is confirmed. In patients under 51 years of age with transformation zone types I and II and no discrepancy between colposcopy, histology and cytology, surgical treatment in the form of laser vaporization could also be performed. The only approved treatment for CIN 2 in transformation zone types II and III is an excision of the cervical transformation zone. The current S3 guideline clearly differentiates the treatment options according to the assessment conditions and transformation zone. However, based on numerous surgical interventions in this patient group, clinical practice and study results, it is evident that the treatment depends significantly on the expertise of the colposcopists and the preferences of the patients [24].

We confirmed that in the active surveillance group, regression was seen in almost 17% of the patients and was statistically significant over the period of observation (*p* < 0.001). Even though our study showed no significant correlation between regression and age, in multiple individual cases, we observed disease regression. We have observed that younger patients are opting for conservative treatment, mainly due to the desire to preserve fertility.

However, in almost 3.6% of the cases, we noted progressive findings and even the development of carcinoma during this time. We have found that even regular check-ups at 6-monthly intervals can still lead to real precancerous stages being overlooked or to developing carcinoma in some cases. This failure of management also occurred in our study. Therefore, we find it essential that even for the patients under active surveillance, regular check-ups and the possibility of progression and, if necessary, surgical treatment, must be discussed.

This can be clarified in a larger cohort with follow-up data available. Further multi-center studies are very important, because the desire to preserve fertility and the demand for conservative therapy is increasing significantly, in part due to the development and spread of reproductive medicine [25]. This may lead to a larger group of patients who would like to undergo surveillance in the presence of HPV-related moderate precancerous lesions in the future.

On the other hand, over of 50% of women diagnosed with CIN 2 in our clinic opted immediately for surgical treatment. In this group, we saw an upgrade of histological findings by 20.4%, which was also described several times previously [26, 27]. This shows how difficult it is to make a histopathological diagnosis of CIN 2 (HSIL) and how high the diagnosis variability is. This explains the upgrading that has taken place in this study group. The other reason for this would be the representativeness of the biopsy; this also shows a certain failure of colposcopy even with very experienced colposcopists. It is crucial to follow up on any lesion to rule out progression [28].

Another aspect to consider is the potential for progression after temporary regression [29]. Previous studies have demonstrated that patients who experience temporary regression ultimately face an elevated risk of recurrence compared to those who have their lesions removed [30]. Wilkinson et al. suggests that long-term follow-up of women who have undergone hysterectomy or cone biopsy is required, as some patients undergo recurrence. This is linked to the reactivation of late infections [31]. However, this is an area that requires further investigation, particularly regarding long-term observations and repeat HPV testing in follow-up for these women.

Our study definitively showed that the spontaneous regression rate of CIN 2 lesions is high, allowing for longer active observation with regular checks and sampling. Accordingly, increasing HPV-genotyping and testing for other biomarkers such as methylation markers will have a significant impact. We feel that good information and patient compliance are essential, because only regular monitoring can detect possible progression at an early stage and prevent the development of carcinoma.

The management of CIN 2 is primarily guided by the expertise and preferences of the colposcopist and the patient. One approach involves regular monitoring with the involvement of a dysplasia specialist, while the other entails a more uncertain prognosis due to the potential necessity for surgical intervention in the event of disease progression.

Our analysis has several limitations. This was a retrospective analysis of a small group of patients (*n* = 187) with CIN 2, which may have limited the power of the study. A further drawback is the limited data available regarding HPV subtyping. We still lack data on the real-life impact of HPV vaccination in this patient group. The registry study is not available in Germany and we are aware that our data, collected in a dysplasia unit at a university hospital, does not provide sufficient evidence to draw conclusions. It is crucial to address the lack of data on vaccination status, which is currently missing from the available medical history. While the expected vaccination coverage rates in our population are likely to be very low, we cannot rule out the possibility that the effects of vaccination or herd immunity may play a role in this population and influence factors such as the distribution of HPV subtypes or regression size. Another weakness of the study is the lack of reference pathology to our histological results. We can only confirm that the diagnosis of CIN 2 is subject to strong intra- and inter-variability, which we see in 20% of the patients where we did not obtain evidence of CIN 2.

### Implications for practice and future research

Although the correlation was not significant in this small study, our data suggest that regression is much more common in younger patient groups. In the future, this may play a role in determining which patients can be monitored and which patients should be treated with surgical intervention. Active surveillance should be based on the patient’s willingness to undergo conservative treatment and a high degree of patience and experience on the part of the treating physician.

We see a statistically significant correlation between HPV 16 positivity and the choice of surgical therapy. HPV-subtyping can impact the selection of treatment.

As an outlook for the future the College of American Pathologists and American Society for Colposcopy and Cervical Pathology classify CIN 2 lesions according to p16 positivity, a marker which predicts the rate of progression. They recommend treating p16 positive lesions and observing p16 negative lesions. Subtyping of HPV and analyzing methylation markers are a promising way to facilitate triage [32].

In our view, the current data support the use of surgical treatment. However, this may change in the future following the inclusion of additional markers to determine the progression of the lesion. This should also be employed to facilitate the transition to personalized medicine, which considers the patient’s preferences and offers the greatest possible safety. HPV-subtyping and other predictive markers can play a very important role in the selection of treatment. It is crucial to differentiate and triage women with CIN lesions according to age, while accommodating patient wishes regarding family planning.

## Supplementary Information

Below is the link to the electronic supplementary material.

Supplementary file1 (DOCX 693 KB)

## Data Availability

No datasets were generated or analysed during the current study.
