# Supplementary material for: The treatment of cervical intraepithelial neoplasia grade 2 (HSIL): between active surveillance and surgery—a 10-year monocentric data analysis
Source: Arch Gynecol Obstet. 2025 Jul 8;312(4):1125–32. doi: 10.1007/s00404-025-08097-1 (PMC12414026; doi:10.1007/s00404-025-08097-1)

Supplement

Fig. 1 Flowchart “CIN 2” study


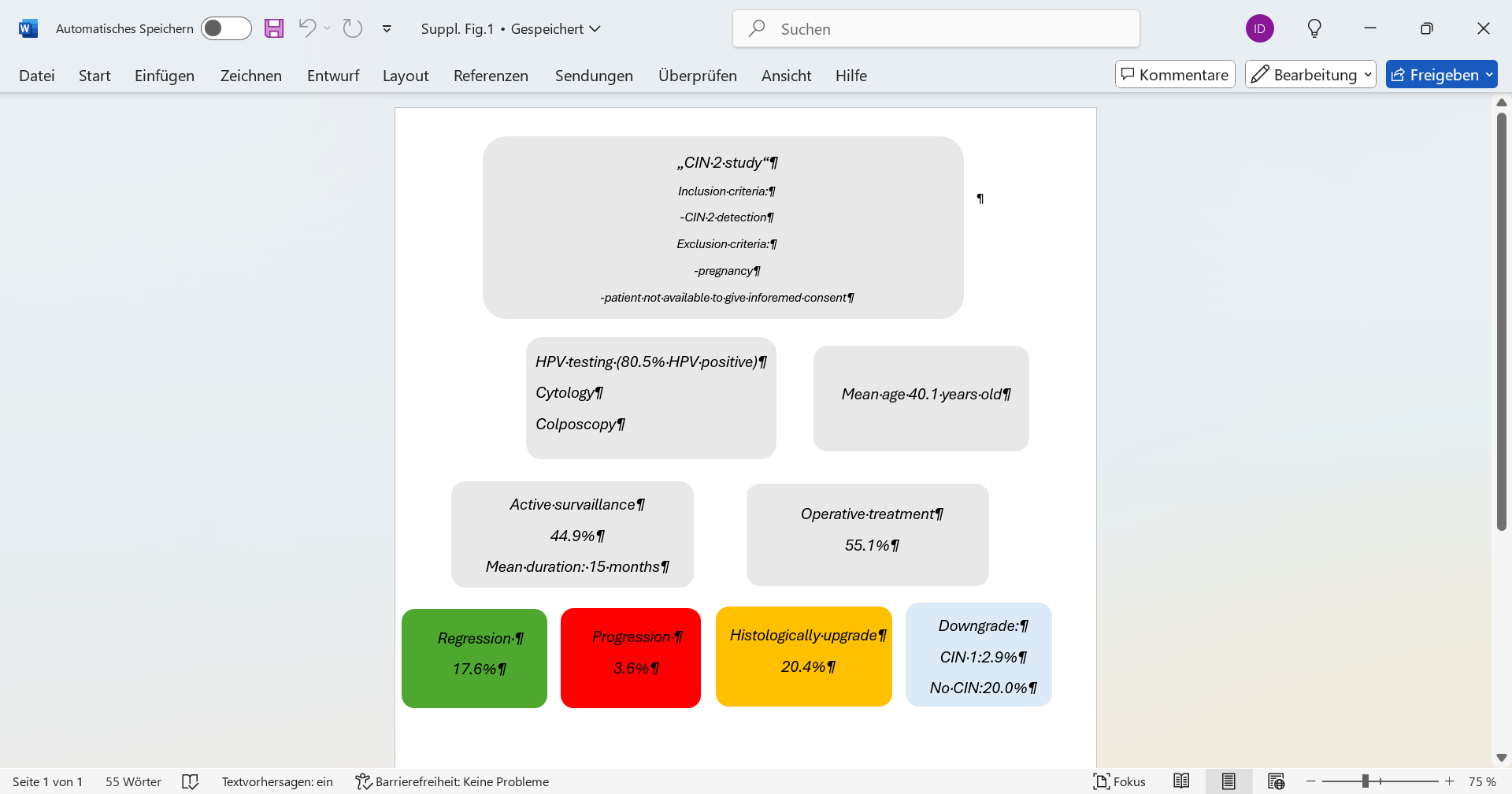


Tab. 1 HPV-Status in the study

| Status of HPV | Prevalence (number of patients) | Percentage |
| --- | --- | --- |
| negative | 20 | 10.3 % |
| positive | 157 | 80.5 % |
| unknown | 10 | 9.2 % |

Fig. 2 Management of CIN 2 in study population

Fig.3 Duration of observation in the active surveillance group


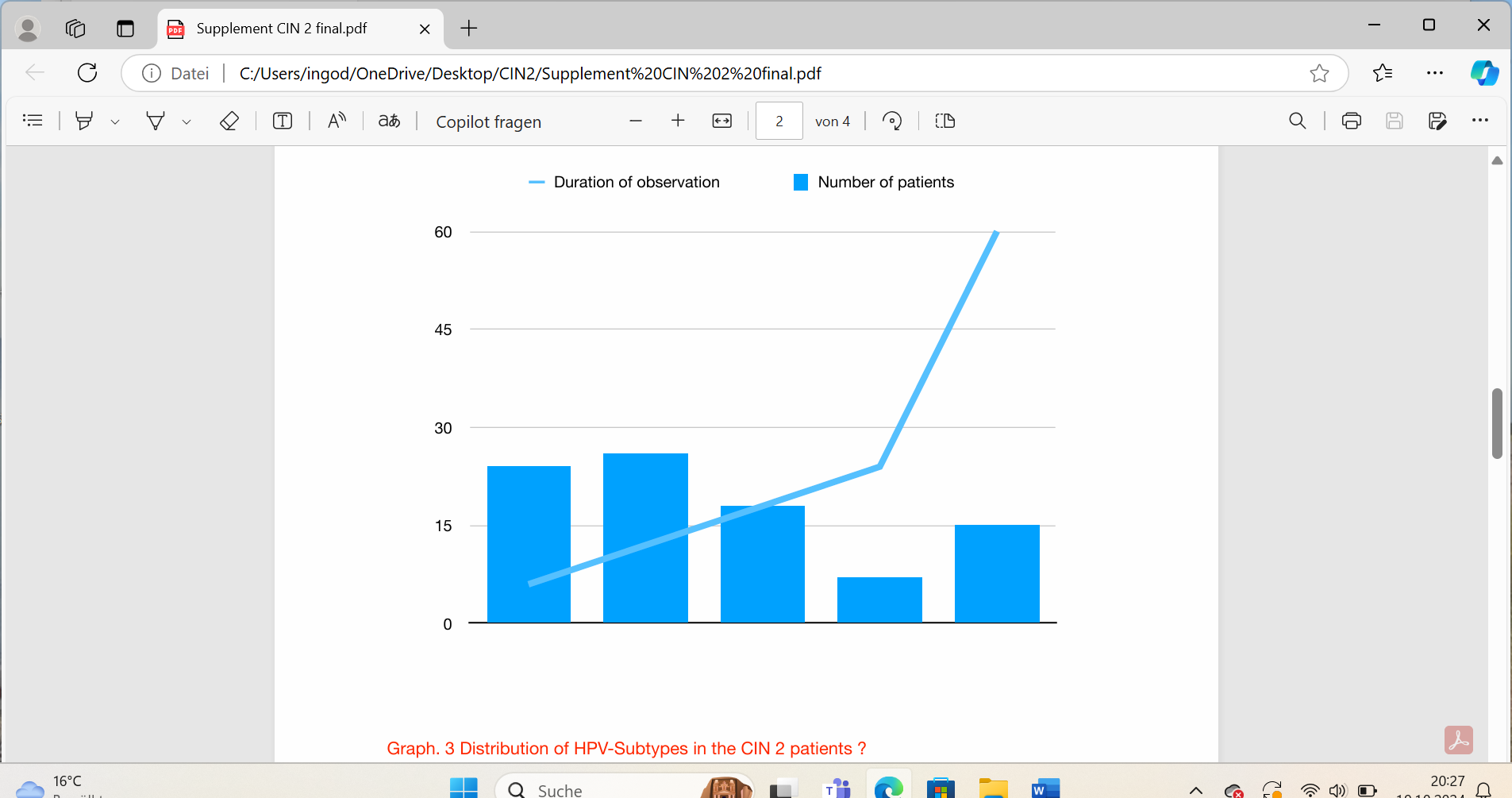


Fig. 4 Age at CIN diagnosis in active surveillance- and operative therapy group

Age
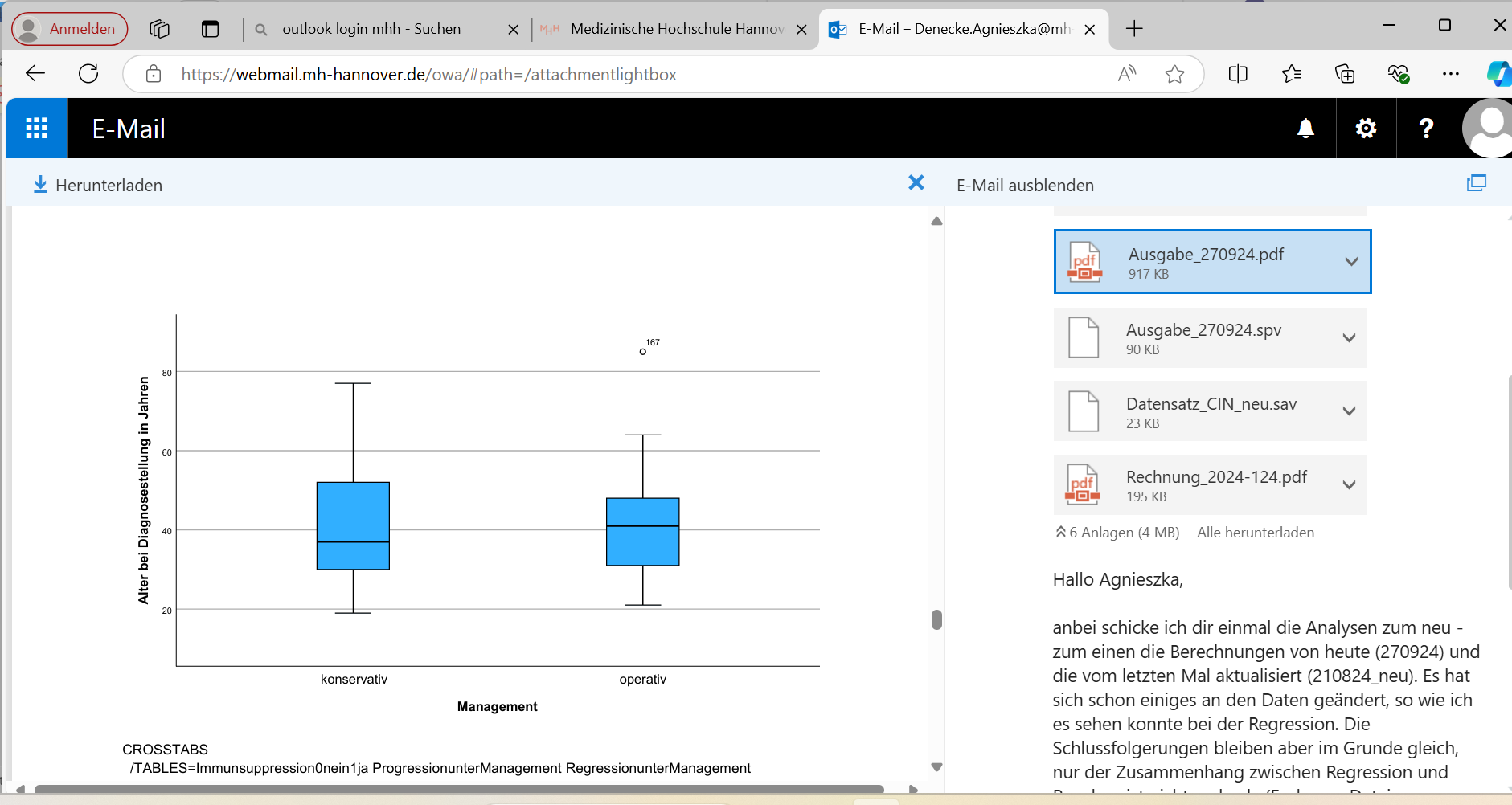


active surveillance operative treatment

Tab. 2 Histologic outcome after operative treatment for CIN 2 diagnosis

| Histologic outcome | Number of patients | Percentage |
| --- | --- | --- |
| CIN 0 | 20 | 19.4 |
| CIN1 | 23 | 22.3 |
| CIN 2 | 39 | 37.9 |
| CIN 3 | 21 | 20.4 |

Fig. 5 Regression and Age


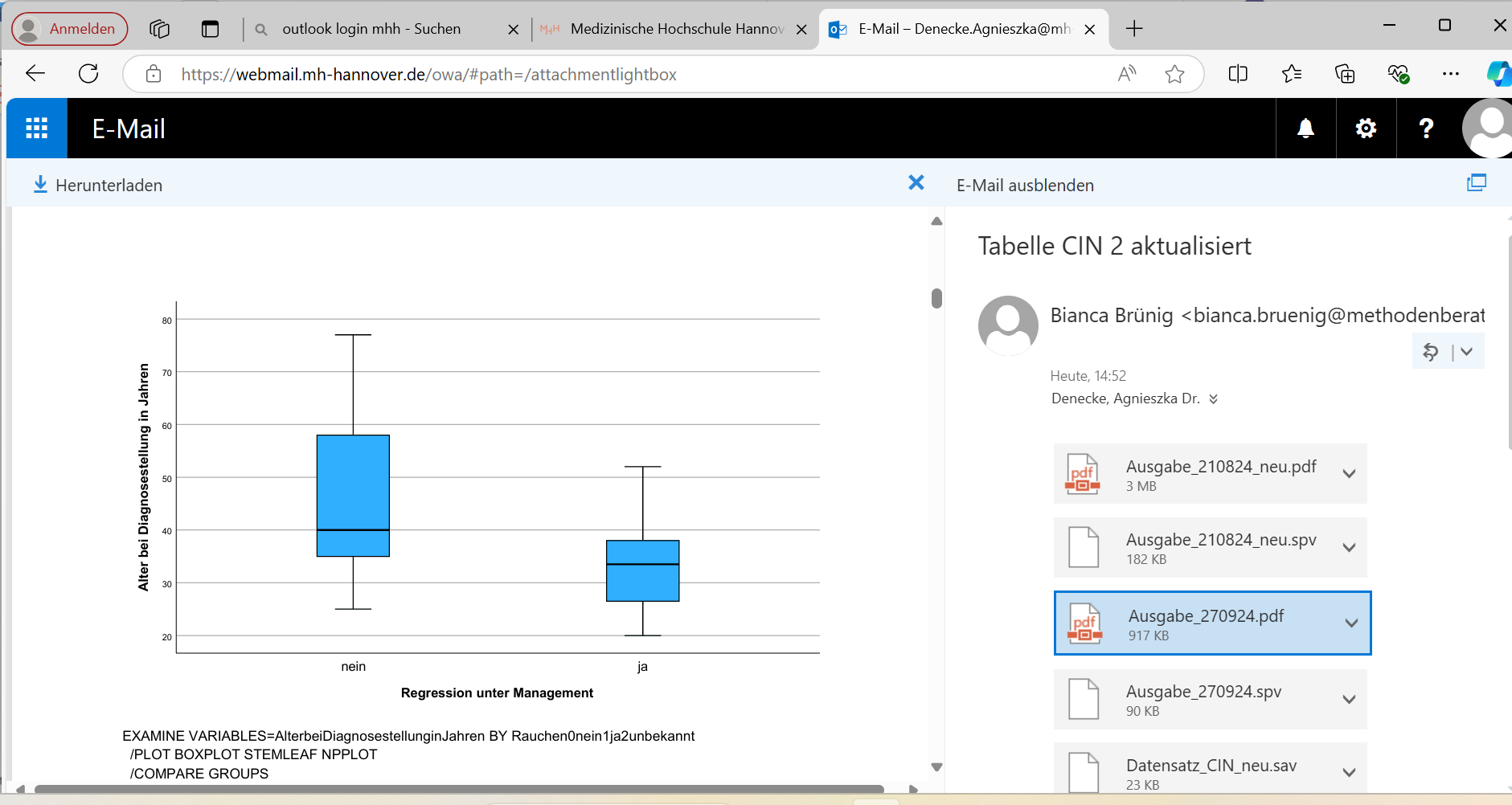

Supplement: Supplementary file 1 — Supplementary file1 (DOCX 693 KB) [file 404_2025_8097_MOESM1_ESM.docx]
